# Past, present, and future options for right ventricular outflow tract reconstruction

**DOI:** 10.3389/fsurg.2023.1185324

**Published:** 2023-06-02

**Authors:** Thierry Carrel

**Affiliations:** Department of Cardiac Surgery, University Hospital Zürich, Zürich, Switzerland

**Keywords:** pulmonary stenosis, pulmonary regurgitation, xenograft, homograft, endogenous tissue restoration, pulmonary valve

## Abstract

The pulmonary valve is the most frequently replaced cardiac valve in congenital heart diseases. Whether the valve alone or part of the right ventricular outflow tract have to be repaired or replaced depends on the specific pathological anatomy of the malformation. Once the decision to replace the pulmonary valve has been made, two options are available: the isolated transcatheter pulmonary valve replacement and the surgical implantation of a prosthetic valve either isolated or in combination with a procedure on the right ventricular outflow tract. In this paper, we will focus on the different past and present surgical options and present a new concept called “endogenous tissue restoration,” a promising alternative to the hitherto existing implants. From a general point of view, neither the transcatheter nor the surgical valvular implants are magic bullets in the arsenal for the management of valvular diseases. Smaller valves have to be frequently replaced because of outgrowth of the patients, larger tissue valves may present late structural valve deterioration, while xenograft and homograft conduits may calcify and therefore become narrowed within unpredictable incidence and interval following implantation. Based on long-term research efforts combining the knowledge of supramolecular chemistry, electrospinning, and regenerative medicine, endogenous tissue restoration has emerged most recently as a promising option to create long-term functioning implants. This technology is appealing because following resorption of the polymer scaffold and timely replacement through autologous tissue, no foreign material remain at all in the cardiovascular system. Proof-of-concept studies as well as small first-in-man series have been completed and have demonstrated favorable anatomic and hemodynamic results, comparable to currently available implants in the short term. Based on the initial experience, important modifications to improve the pulmonary valve function have been initiated.

## Introduction

A considerable number of patients suffering from pulmonary stenosis (PS), pulmonary atresia, Fallot tetralogy (TOF), truncus arteriosus, double outlet with PS, transposition of the great artery (TGA) with PS requiring a Rastelli procedure, as well as those with even more complex anomalies may require multiple procedures on the pulmonary valve and/or right ventricular outflow tract (RVOT). There are multiple reasons for this: normal outgrowth (especially when small implants have been used in neonates and smaller children), degeneration of biological tissue with severe calcifications, and consecutive valvular stenosis or anastomotic narrowing following homograft and xenograft implantation. This makes the pulmonary valve (PV) the most frequently replaced cardiac valve in congenital heart diseases (1). Nonetheless, any strategy to preserve the pulmonary valve at initial surgery is of course welcome (2), and procedures without preservation of the pulmonary valve have been used as well.

However, options for pulmonary valve and/or RVOT replacement are preferred. Among several techniques, surgical and percutaneous tissue valves can be implanted, with limited durability being the major limitation of this technology. Xenograft tissue tends to degenerate and become calcified with time; this is frequently observed in the adult population where such valves are frequently implanted in the aortic position, but the same is true for the right-heart position, even though the mechanical stress is smaller due to lower blood pressure (3, 4). Therefore, independent from the position it has been implanted, re-intervention is frequent in patients who receive a biological valve prosthesis. Basically, there are no major differences expected following surgical or percutaneous tissue valves. With increasing children's survival at the initial operation and improved outcome in the long-term, a majority of these patients reach adulthood and may require additional procedures up to their fifth or sixth life decade. Therefore, considerations concerning outgrowth, hemodynamic performance, durability of the implanted conduits and valves, as well as operative challenges are of great interest.

## The past and the present

### Valveless option

Even though there are multiple possibilities to replace the pulmonary valve, occasional reports have been published on cases where a valveless conduit was used (5). From the history, transannular pericardial patch without a valve or with remnants of a valve only has been used years ago in patients with Fallot tetralogy; most recently, some authors have reported acceptable outcomes with a similar strategy (5).

### Prosthetic tissue and mechanical valves

When pulmonary valve replacement is planned, the most discussed topic is still which type of implant should be used in which age category? In the vast majority, a tissue valve is considered the best choice but there is a small subset of older patients in whom a mechanical valve may be used as an alternative, especially when these patients need long-term oral anticoagulation for other reasons.

Current tissue valves are based on animal-derived glutaraldehyde-fixed pericardial (bovine, porcine, or even equine) tissue, and much more rarely on the native valve of the animal. The construction of such valves alone includes several issues, such as access to the animal material; the reproducibility and consistency of material performance; manufacturing challenges; and finally chronic inflammatory response, tissue thickening, and/or subclinical thrombosis of the valve leaflets. Some of these issues may be accelerated in children because of the higher hemodynamic performance and the more pronounced competence of the immune system. As a consequence, redo-procedures (percutaneous or surgical ones) are often required to resolve the problem of stenosis and/or regurgitation of the previous implants (6–8). This is the reason why research for alternative solutions has to be heavily supported.

Data available on outcomes following the implantation of a mechanical valve in pulmonary position are scarce (9–11). In addition, the results are inconsistent when not contradictory. In smaller series, dysfunction of the mechanical valve was observed in up 35% of the patients as early as 6 months postoperatively (12). Longer follow-up is limited, and for those patients surviving without complications, mechanical pulmonary valve implantation is usually maintained on an aggressive regime of oral anticoagulation. This is the reason why the subset of patients that may benefit from a mechanical valve in the right ventricular outflow is estimated to be 1%–2%.

### Autologous pericardial valved conduit

After extensive experience with the use of autologous pericardium for reconstruction of the right ventricular outflow tract, Kreutzer et al. developed the concept of a fresh autologous valved pericardial conduit to connect the venous ventricle with the pulmonary artery (13, 14). The objective was to reproduce the excellent long-term results of untreated autologous pericardial patches without a valve. The technique includes a pericardial patch for the RVOT and a bicuspid pericardial valve that is fixed within the patch. The creation of the autologous pericardial is somewhat similar to what has been described by Ozaki et al. for the aortic valve (15, 16). It is a welcomed technique in case no alternative implant material is available, especially in emerging countries. The results reported in a series of more than 130 patients with different diagnoses were excellent (14). They were aged from 15 days to 24 years, and the average size of the conduit was 15 mm. During an observational interval up to 19 years, there were 12 reoperations with only 3 conduit replacements. Freedom from conduit replacement at 5, 10, and 15 years was 90%, 81%, and 77%, respectively. Similar favorable results have been reported by others.

### Xenograft pericardial conduits

The Contegra bovine jugular conduit showed originally considerable promise as a pulmonary valve and right ventricular outflow tract replacement implant, especially in smaller children (17–23). This conduit is available from 12 to 22 mm in size, does not require oral anticoagulation, does not shrink, and maintains reliable valve competence in a high percentage of patients. We have also had extensive experience with this conduit, starting from 2000. The overall intraoperative, early postoperative, and midterm experience was a very satisfactory one. Nonetheless, this type of implant also presented with structural degeneration after an average follow-up of 6–10 years in our experience with a significant proportion of patients requiring either a re-intervention (usually implantation of a transcatheter Melody pulmonary valve) or a surgical re-exploration with the complete exchange of the conduit (24). Those patients presenting with outgrowth had almost always some degenerative changes at the time of explantation. In patients with extremely calcified xenograft conduits (but also in those with circumferentially calcified homografts), the peel technique is a valuable option that consists of preserving the sides and posterior half of the previously placed conduit while a prosthetic roof is placed over the conduit remnant. Cervantes-Salazar et al. found this technique safe and easy to teach residents (25).

### Stentless porcine aortic root

In 2009, Hawkins et al. reported on their experience using the Freestyle 19 mm conduit and the Prima for all sizes other than 19 mm (26). This conduit was initially developed for reconstruction of the aortic root but was also found to be a good option for the right ventricular outflow tract. The implantation technique is exactly the same as for other conduits, but since the porcine aortic root is rather short, the use of a bovine pericardial patch as a gusset to close the right ventricle below the plane of the neo-pulmonary valve is rather common.

Kuo et al. analyzed all non-Ross RVOT reconstructions using the Freestyle root, especially survival and re-intervention, either by surgery, transcatheter valve implantation, balloon valvuloplasty, or bare metal stent placement (27). Out of 182 patients, 163 patients were identified for a follow-up. Median age was 12.2 years, median weight was 39.0 kg, and the median body surface area was 1.23 m^2^. Of the patients, 57% had tetralogy of Fallot. Median follow-up was 5.4 years and 38 patients (23%) required re-intervention. The rate of freedom from re-intervention decreased from 93.2% at 5 years to 48.4% at 10 years. Age <10 years, weight <39 kg, and body surface area <1.2 m^2^ at the time of valve placement were significantly associated with need for earlier re-intervention. The longevity of this implant was found to be comparable to that published for homografts and other tissue valves.

### Pulmonary and aortic homografts

Aortic and later pulmonary homografts were the first “anatomical” valved conduits that have been used in the treatment of more complex congenital heart diseases almost 50 years ago, especially when the right ventricular outflow tract has to be replaced. While aortic homografts are robust, they may present with severe degeneration in the midterm follow-up, which means around 8–12 years. In these cases, the major problem is issued from the valve that becomes stenotic while the vascular wall of the aortic homograft becomes heavily calcified and may cause technical difficulties when a complete replacement is necessary.

Pulmonary homografts have been increasingly used for RVOT reconstruction in congenital surgery as the first approach, but also when more complex surgery has to be performed in the re-operative setting (28).

Aortic and pulmonary homografts demonstrate reasonable outcomes in terms of hemodynamics, very low thromboembolic risk, and a better resistance to infection than mechanical ones or tissue. Nonetheless, there are some drawbacks associated with homografts as far as the restricted availability is concerned but also issues with the limited durability with about 30%–40% of homografts are still functional after 20 years. As expected, early degeneration and/or problems caused by outgrowth have been observed more frequently in pediatric than in adult patients as degradation is even faster.

After the implantation of a pulmonary homograft, regurgitation may develop but is rarely the main reason for a redo-operation. Obstruction of the pulmonary homograft caused by severe calcification of the valve is a much more frequent reason for a reoperation/re-intervention. In smaller patients, in whom the pulmonary homograft can be oversized, a substantial prolonged durability may be observed until a repeat intervention/surgery is necessary. Another interesting topic has been the implantation of blood group–compatible homografts to increase the durability (29). Homografts are still considered by many surgeons as the first choice for pulmonary valve replacement (PVR) in patients suffering from TOF and in those in whom a Ross procedure is performed even though reoperation or re-intervention are rather common in the long term (30, 31).

Recently, an interesting study analyzed the outcome of pulmonary homografts in a well-defined group of 26 adult patients (mean age 30 ± 8 years) suffering from tetralogy of Fallot with median follow-up of 17 years (31). Main study outcomes were survival and hemodynamic parameters like pulmonary regurgitation, right ventricular (RV) end-diastolic volume, RV ejection fraction, left ventricular (LV) end-diastolic volume, LV ejection fraction assessed by MRI, and New York Heart Association functional class. Two patients needed replacement of the homograft at 24 and 39 months after PVR. The indication in both patients was the recurrence of severe homograft regurgitation with important RV dilatation. After a follow-up of 17 years, 23 out of 26 patients (89%) were alive without redo PVR. There was no significant deterioration of hemodynamic function or functional class in the remaining patients.

Another less frequently used option is the implantation of a femoral vein homograft in the right ventricular outflow tract position (32, 33).

### Polytetrafluoroethylene monocusp pulmonary valve reconstruction

Reconstruction of the right ventricular outflow tract using a monocusp neo-pulmonary valve from polytetrafluoroethylene (PTFE) material may create a competent and non-stenotic pathway between the right ventricle and the pulmonary artery. This concept helps obtain rather physiological conditions at the end of the operation helping right ventricular mechanics closely resemble those of a normal outflow tract or those following a simple valve reconstruction. There is sufficient evidence that this approach may provide midterm remodeling advantages compared to procedures where the pulmonary valve is not maintained. Turrentine et al. published more than 10 years ago on the advantages of such an approach, which effectively avoids pulmonary insufficiency in the early postoperative course but also at midterm, without any evidence of stenotic dysfunction in the long-term (34). This technique has been described in detail earlier, and it is reproducible and quite easy to learn.

While the literature has been inconclusive regarding the short-term clinical benefits of monocusp RVOT reconstruction, there has been acceptable long-term series with both monocusp and bicuspid PTFE valves. The series of Turrentine comprises 196 patients that demonstrated only mild to moderate insufficiency in 58% of the patients at a follow-up of 10 years and no stenosis (34).

### Polytetrafluoroethylene bicuspid pulmonary valve implantation

Following reasonable experience with monocusp PTFE RV to pulmonary artery (PA) reconstruction regarding the function of the valve and the durability of the leaflet, Quintessenza et al. reported on a bicuspid valve implantation made of two PTFE leaflets (35). The initial series included 41 patients and demonstrated an improvement with regard to pulmonary valve insufficiency, right ventricular end-diastolic dimensions, and clinical conditions over a follow-up period of 18 months—which is of course not long enough for definitive evaluation of this type of PTFE valve. Later, the same group reported on 110 patients with a follow-up up to 8 years, including three explants because of immobile and calcified PTFE leaflets. In the remaining patients, the results were promising.

### Simplified standardized trileaflet polytetrafluoroethylene valved conduit

More than 10 years ago, the use of an RV-PA conduit made of expanded polytetrafluoroethylene has been reported by Japanese surgeons with promising long-term stability, when valve function was concerned (36). However, the construction of such conduits with bulging sinuses is still considered critical to be reproduced by less experienced groups. Tocharoenchok et al. reported on a simplified standardized technique to facilitate the construction of the conduit but considered the surgical technique of implantation as important as the design itself, since kinking or folding may lead to pathological pressure gradients and therefore contribute to premature degeneration (37). The authors described a detailed technique for constructing such a valved conduit recently. Implantation starts with the distal anastomosis first, performed close to the distal attachment site of the leaflets in the conduit. The proximal anastomosis is performed as a second step following precise trimming of the conduit to avoid any geometric distortion. This group implanted 100 conduits of sizes 16–24 mm between 2018 and 2022. The median follow-up was 589 days (up to 897 days); echocardiography was performed between 6 and 12 months and revealed a peak gradient of 18 mmHg with mild or less regurgitation in all patients. No reoperation was performed in this limited follow-up period. The authors considered this simplified method of making a trileaflet conduit as an excellent alternative to homo- and xenograft conduits because of its availability on the shelf.

The Kyoto University of Medicine group has recently reported their experience with 1,776 patients (median age 4 years, ranging from 3 days to 67 years; and median weight 13 kg, ranging from 1.8 to 90 kg) who received an expanded polytetrafluoroethylene (ePTFE) conduit with bulging sinuses and a fan-shaped valve for RVOT reconstruction; a 0.1-mm-thick PTFE membrane used for the pulmonary valve leaflets (38). Following a median observational interval of 3 years, they reported a re-intervention rate of 16%, including an explantation rate of 11%. The main reason for explantation was somatic growth while endocarditis of such ePTFE conduits was infrequent (4%). The most frequent reason for catheter intervention was peripheral pulmonary artery stenosis in 4%. At the last echocardiography, pulmonary valve function better than mild regurgitation was observed in 88% of the patients.

Neointimal proliferation with subsequent calcification, a rather frequent finding following xenograft or homograft implantation, was not an issue in this large series. A very acceptable valvular function was observed following implantation of large-size ePTFE conduits but exactly when using such larger conduits, special care should be directed to the optimal length of the conduit since a too long conduit may compress the peripheral pulmonary artery and lead to pulmonary stenosis. Some authors prefer to use a Dacron graft as a conduit to restore the RV to PA continuity but PTFE material to create the neo-pulmonary valve within this graft (36).

### A pulmonary valve from the right atrial appendage

Recently, some authors from Iran have introduced a completely new technique to create a bicuspid pulmonary valve out of the native tissue of the right atrial appendage (39). Their initial experience has been confirmed by encouraging own midterm results but has been confirmed by others so far (40). The procedure was originally designed for patients suffering from Fallot tetralogy; as the preliminary results were encouraging, the authors used it for different types of RVOT pathologies, including repair of truncus arteriosus, pulmonary atresia, and Nikaidoh procedure. With increasing practical experience, the authors were able to demonstrate that practically all appendages may be used for a valve construction; however, those appendages with a width tall and a half times their height were the most suitable. Reconstruction of the pulmonary valve is performed after intracardiac correction, like closure of a ventricular septal defect (VSD). As described by the authors, the anulus width should be approximately half of the circumference of an average proper size according to the patient's body surface area. While the RVOT is open, the anterior half of the RV-PA continuity is constructed using a patch of xeno-pericardial tissue.

The large majority of patients who received this type of right atrial appendage (RAA) valve had no or trivial pulmonary regurgitation during midterm follow-up; as it is made up of “living” tissue, the atrial appendage valve may have some potential to grow.

## The future

### Polymer scaffolds with the potential for endogenous tissue restoration

Endogenous tissue restoration (ETR) is a disruptive technology that has the following advantages: fully controllable manufacturing, less issues in biocompatibility (less leaflet thickening/thrombosis), availability from the shelf and dry storage, reduced or even no need for anticoagulation, and hopefully improved durability. In addition, potential growth once the polymer has disappeared and endogenous tissue has been built up is a significant new characteristic of this technology.

ETR is another very attractive concept that relies on the fact that bioabsorbable material (a polymer as scaffold) may be replaced by own body tissue with all advantages of avoiding foreign body material. ETR combines three scientific disciplines: supramolecular chemistry, electrospinning, and regenerative medicine (41–50). Nowadays, different tunable supramolecular polymers are available and they are characterized by different degrees of mechanical strength and rates of bioabsorption. Using the technique of electrospinning, unique bioabsorbable matrices can be created in which the polymers are assembled in a random fashion, creating a matrix that can be easily penetrated by endogenous cells, such are red cell, platelets, macrophages, fibroblasts, and myofibroblasts.

The “endogenous tissue restoration” process can be divided into three important steps: (1) implantation of the prosthesis serving as scaffold, (2) neo-tissue formation, and (3) functional restoration with resorption of the scaffold. The critical balance between tissue formation and implant absorption is the key for success of this technology since the patient's tissue has to undertake the function of the implant when it has completely disappeared. From an anatomical and physiopathological point of view, ETR is defined as the replacement of the absorbable polymer leaflet and conduit by a patient's own native cells that infiltrate the matrix and trigger a cascade of physiologic events with gradual replacement by native tissue.

**Figure 1:**
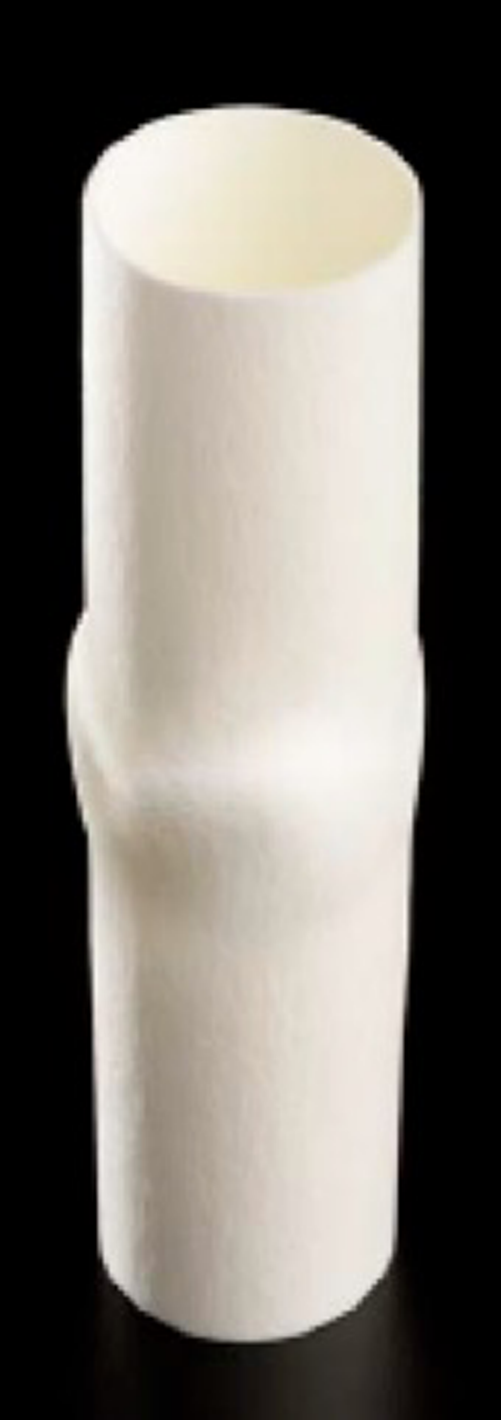
The polymeric tube-valved conduit (with courtesy from Xeltis, Eindhoven, Netherlands).

**Figure 2:**
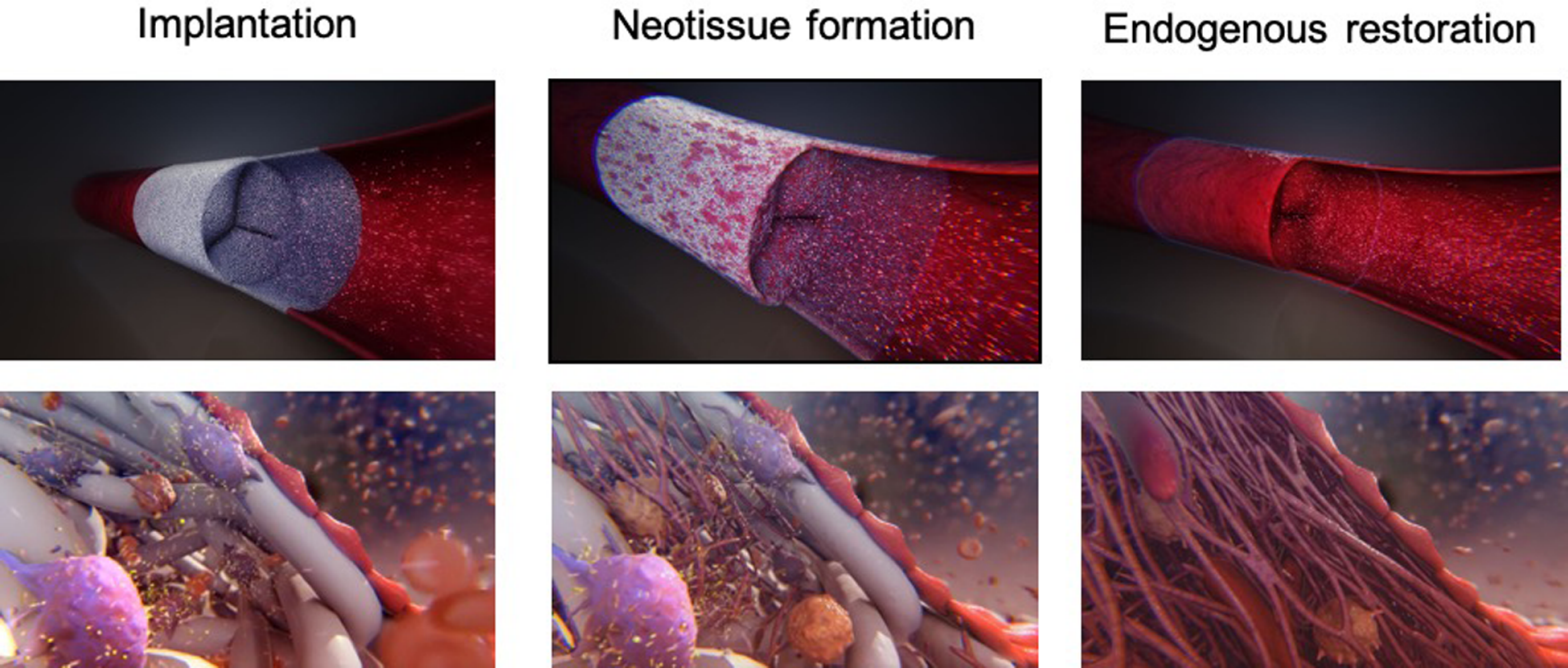
Schematic representation of the endogenous tissue restoration process in three phases: soon after implantation (left), during neo-tissue formation (middle), and following most complete endogenous restoration (right). Recipient cells attach on the scaffold, followed by conjunctive tissue formation and endothelialization of the inner layer (with courtesy from Xeltis, Eindhoven, Netherlands).

**Figure 3:**
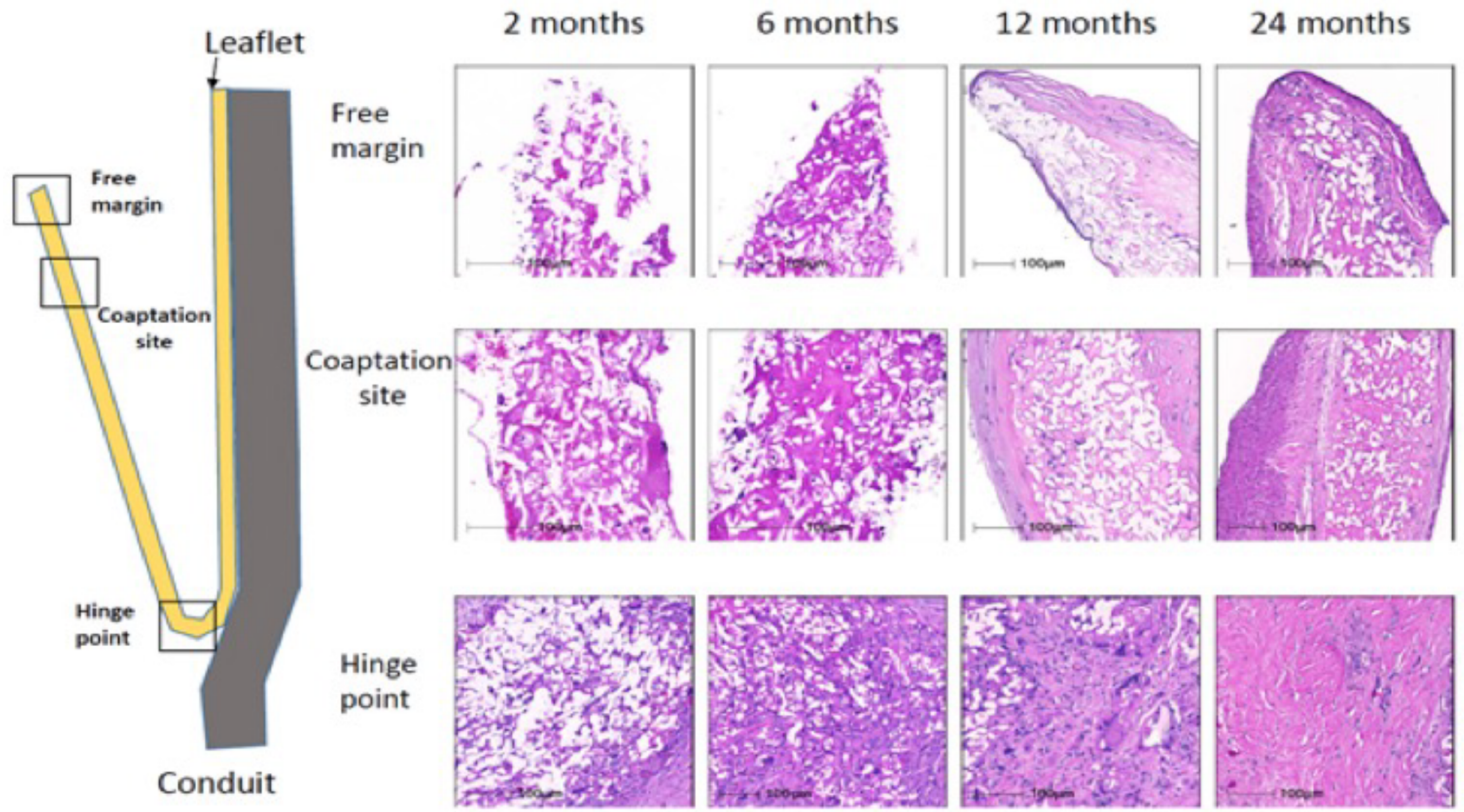
Serial changes in leaflet histology of novel bioabsorbable pulmonary valved conduit overtime [reproduced with permission from Bennink et al. (48)].

**Figure 4:**
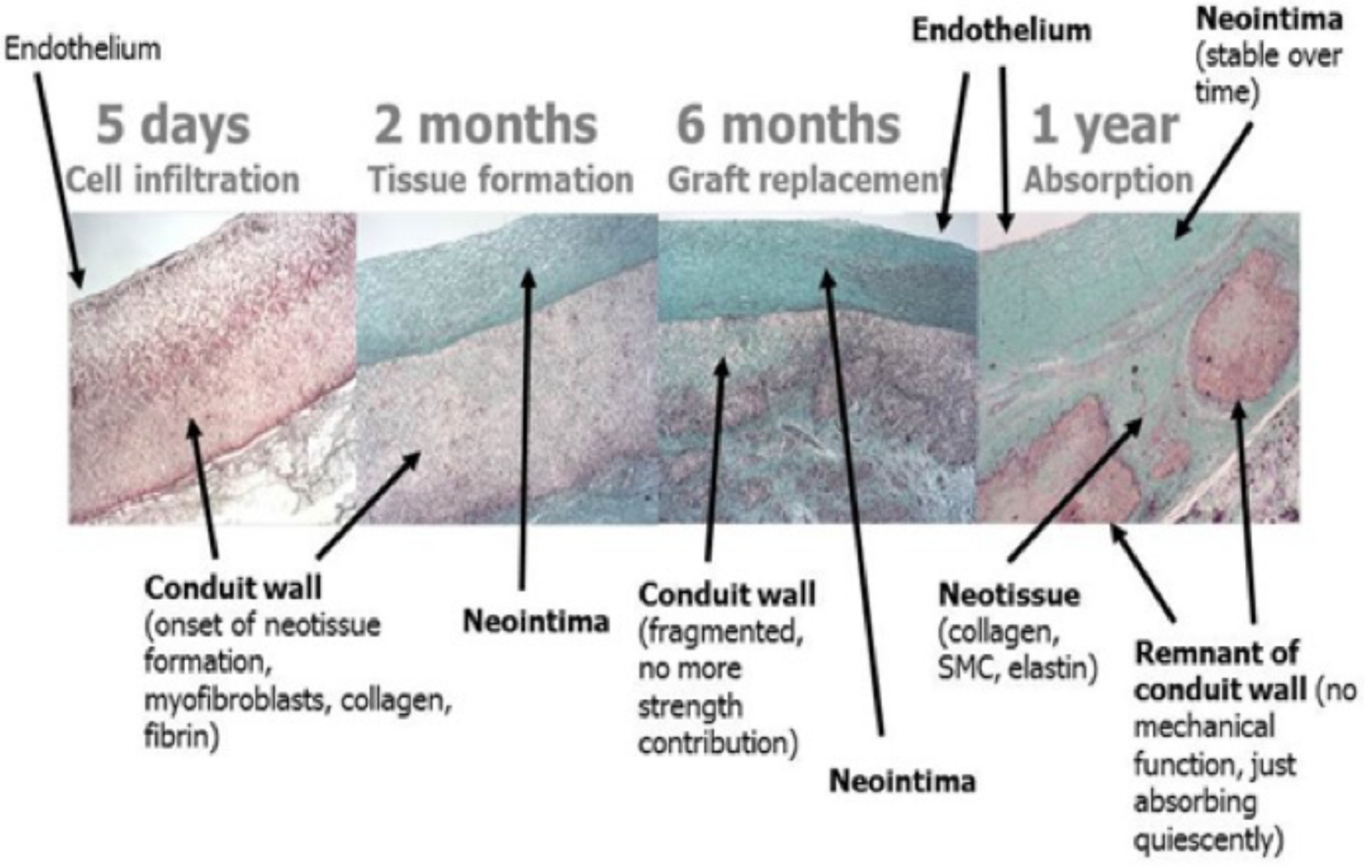
Serial changes of a bioresorbable pulmonary valved conduit implanted in a sheep (preclinical Xeltis valve study) [reproduced with permission from Bennink et al. (48)].

Grossly, the conduit looks like a PTFE prosthesis (Figure 1). As absorption begins, the leaflets and the conduit are infiltrated by inflammatory cells, releasing growth factors, promoting smooth muscle cells infiltration and matrix production (proteoglycans, collagen with focal elastic tissue) (Figure 2). Figure 3 shows the ETR in a pulmonary valved conduit at three different levels of the leaflet, at the free margin, at the coaptation site, and at the hinge point. Over a period of 24 months, there is a leaflet collagen replacement with leaflet matrix absorption. This pulmonary valved conduit showed encouraging results in a sheep model up to 24 months (Figure 4) and may represent a significant improvement over the current conduits available for children with congenital heart diseases who need reconstruction of the right ventricular outflow.

**Figure 5:**
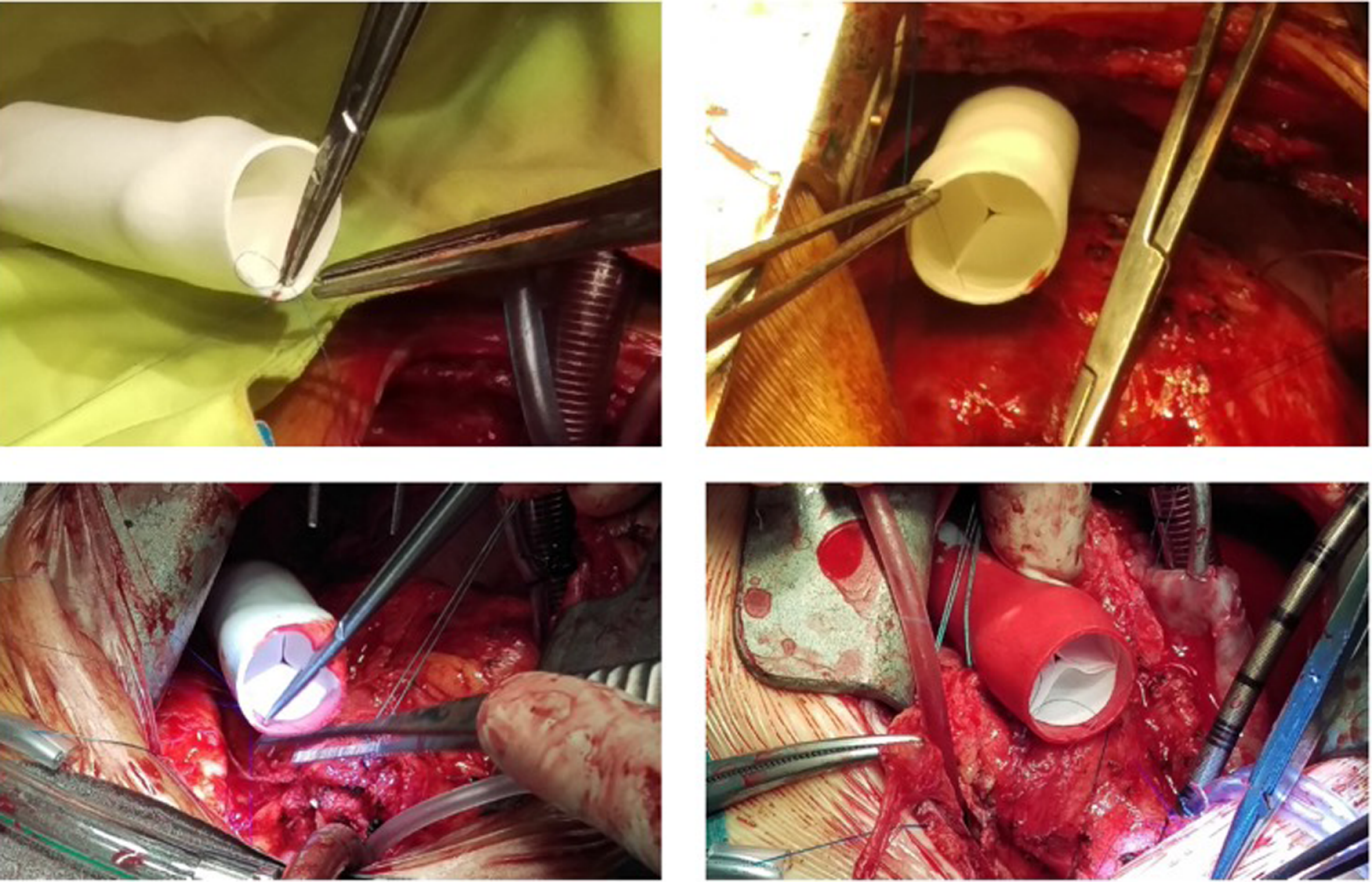
Xeltis pulmonary valved conduit implantation in the RVOT of a patient. RVOT, right ventricular outflow tract.

From the preliminary clinical experience, five children (4–12 years) who received a 20 mm extracardiac conduit between the inferior vena cava and the pulmonary artery as part of the Fontan procedure have been reported. They had no device-related adverse event and MRI demonstrated good hemodynamics, anatomical, and functional stability of the graft (48). Following this initial study, an EU clinical feasibility study on the Xeltis pulmonary valved conduit was initiated as well and 12 patients (2–12 years, 17–43 kg) were enrolled in a prospective, nonrandomized, open-label study to assess the safety of this new pulmonary valved conduit (14). The size of the conduits was 16 (*n* = 5) or 18 (*n* = 7) mm, and technical success was achieved in all patients. Figure 5 shows the intraoperative finding during implantation of the Xeltis conduit. Longer term data from the study are still being collected. Diagnoses were pulmonary atresia with VSD (*n* = 4), tetralogy of Fallot (*n* = 4), common arterial trunk (*n* = 3), and transposition of the great arteries with VSD and pulmonary stenosis (*n* = 1). All had had a previous surgery, including prior RVOT conduit implantation in six. At 24 months, none of the patients required surgical re-intervention; 9 of the 12 are in NYHA functional class I and 3 in NYHA class II. None of the conduits have shown evidence of progressive stenosis, dilation, or aneurysm formation. Residual peak gradient of >40 mmHg was observed in three patients, most probably caused by kinking of the conduit at implantation in one and distal stenosis in the peripheral pulmonary arteries in two patients. Five patients developed pulmonary valve insufficiency; the most common mechanism was prolapse of at least one of the valve leaflets. This has led to design improvement (geometry, thickness) of the valve leaflets.

In summary, the potential benefits of the Xeltis technology can be described at three levels: (1) acute benefit, since the restorative material allows for profile reduction of the valve and allows for dry storage without glutaraldehyde; (2) manufacturability, as scalable good manufacturing practice in polymer production is possible and there is no need for sourcing of animal tissue. In addition, less chronic inflammatory process is expected, since there will be no response to foreign body such as fixate tissue.

**Figure 6:**
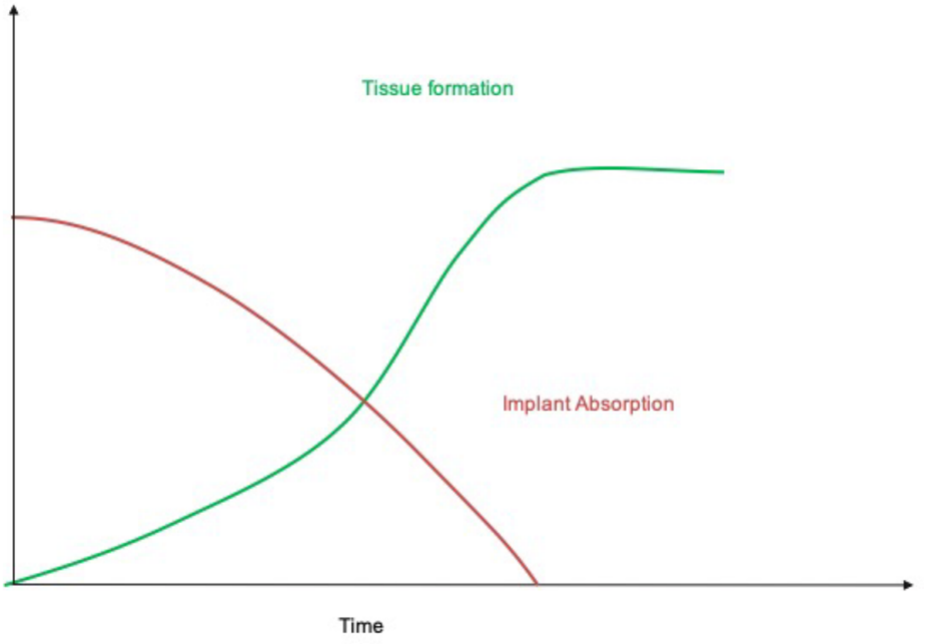
Schematic representation of the process of endogenous tissue restoration over time. The implant is gradually absorbed while neo-tissue formation replaces the polymeric scaffold (with courtesy from Xeltis, Eindhoven, Netherlands).

In conclusion, XELTIS platform is a synthetic, resorbable cardiovascular device that, after implantation, enables generation of a heart valve or blood vessel with the patient's own tissue. This restorative technology has the potential to redefine heart valve replacement, especially in children and younger adults. The tunable characteristics offers the possibility to rather precisely determinate the process of implant absorption and neo-tissue formation (Figure 6).

## Conclusions

Pulmonary valve replacement or right ventricular to pulmonary artery conduit implantation belongs to the most frequent late intervention in congenital heart surgery. To ensure the most optimal treatment and the choice of the most convenient implant (Table 1), a collaborative approach between pediatric cardiology, invasive cardiology, and cardiac surgery is required (51).

**Table 1:** Summarizes the main characteristics of the potential devices that may be used for PA/RVOT repair and/or replacement

	Maintained PV function	Foreign material	Autologous material	Structural degeneration	Complex procedure	Anticoagulation	Capacity to grow	Results available	Large experience	Favorable results	Availability on the shelf
Valve excision	−	−	+	−	−	−	±	+	+	±	
Mechanical prosthesis	+	+	−	−	−	+	−	+	−	±	+
Biological prosthesis	+	+	−	+	−	−	−	+	+	+	+
Autologous pericardial valve conduit	±	−	+	+	+	−	−	+	−	±	−
Xenograft	+	+	−	+	−	−	−	+	+	±	+
Stentless porcine aortic root	+	+	−	+	−	−	−	+	−	+	+
Homograft	+	+	−	+	−	−	−	+	+.	+	−
PTFE pulmonary valve construct	±	+	−	−	+	−	−	±	−	±	−
Right atrial appendage pulmonary valve	+	−	+	?	+	−	+	±	−	?	−
Endogenous tissue restoration	+	±	+	?	−	−	+	±	−	±	+

PTFE, polytetrafluoroethylene.

Once all anatomical and functional alterations have been diagnosed, decision-making is needed toward preservation or not of the pulmonary valve, if yes: surgery or a transcatheter approach (52) will have to include the following considerations:

- Single problem of the RVOT/PV or a more complex pathology?
- Risk of both approaches?
- Potential advantages of a palliative treatment until the patient gets bigger?
- Presence or not of infection, pseudo-aneurysm, arrhythmia, risk of left main compression?

Depending on the fact of the current RVOT anatomy (native pulmonary valve dysfunction, dysfunction of a previously implanted tissue valve, homograft or xenograft conduit), the question on how to proceed—surgical intervention vs. transcatheter valve implantation?—will become actual. The position of a conduit (orthotopic or extra-anatomic) is of utmost importance when the relationship with the left main stem but also with the posterior surface of the sternum is concerned. Furthermore, the presence of additional hemodynamic pathologies, arrhythmias, and also the function of the tricuspid valve as well as the fact if the surgery will be a redo or not are important factors in the decision-making process. Nevertheless, a personalized procedure (surgical, transcatheter, hybrid through the right ventricle) can be offered depending of the clinical, anatomical and functional problem. In this context, tissue engineering and as part of it ETR becomes a new dimension and has to be followed closely (53).
